# Quantitative Spectrochip-Coupled Lateral Flow Immunoassay Demonstrates Clinical Potential for Overcoming Coronavirus Disease 2019 Pandemic Screening Challenges

**DOI:** 10.3390/mi12030321

**Published:** 2021-03-18

**Authors:** Kai-Feng Hung, Chih-Hsing Hung, Chitsung Hong, Szu-Chia Chen, Yi-Chen Sun, Jyun-Wei Wen, Chao-Hung Kuo, Cheng-Hao Ko, Chao-Min Cheng

**Affiliations:** 1 Division of Translational Research, Department of Medical Research, Taipei Veterans General Hospital, Taipei 112, Taiwan; g38913008@gm.ym.edu.tw; 2 Department of Dentistry, School of Dentistry, National Yang-Ming University, Taipei 112, Taiwan; 3 Department of Pediatrics, Kaohsiung Medical University Hospital, Kaohsiung Medical University, Kaohsiung 807, Taiwan; pedhung@gmail.com; 4 Department of Pediatrics, Kaohsiung Municipal Siaogang Hospital, Kaohsiung Medical University, Kaohsiung 807, Taiwan; 5 Spectrochip Inc., Hsinchu 300, Taiwan; cthong23@gmail.com (C.H.); wenjyunwei@gmail.com (J.-W.W.); 6 Division of Nephrology, Department of Internal Medicine, Kaohsiung Medical University Hospital, Kaohsiung Medical University, Kaohsiung 807, Taiwan; scarchenone@yahoo.com.tw; 7 Department of Internal Medicine, Kaohsiung Municipal Siaogang Hospital, Kaohsiung Medical University, Kaohsiung 807, Taiwan; 8 Department of Ophthalmology, Taipei Tzu Chi Hospital, The Buddhist Tzu Chi Medical Foundation, New Taipei City 231, Taiwan; yichensun.tzuchi@gmail.com; 9 Division of Gastroenterology, Department of Internal Medicine, Kaohsiung Medical University Hospital, Kaohsiung Medical University, Kaohsiung 807, Taiwan; 10 Graduate Institute of Automation and Control, National Taiwan University of Science and Technology, Taipei 106, Taiwan; 11 Institute of Biomedical Engineering, National Tsing Hua University, Hsinchu 300, Taiwan

**Keywords:** coronavirus disease 2019 (COVID-19), severe acute respiratory syndrome coronavirus-2 (SARS-CoV-2), lateral flow immunoassay, reflectance spectrum, detection limit

## Abstract

As coronavirus disease 2019 (COVID-19) continues to spread around the world, the establishment of decentralized severe acute respiratory syndrome coronavirus-2 (SARS-CoV-2) diagnostics and point-of-care testing is invaluable. While polymerase chain reaction (PCR) has been the gold standard for COVID-19 screening, serological assays detecting anti-SARS-CoV-2 antibodies in response to past and/or current infection remain vital tools. In particular, lateral flow immunoassay devices are easy to produce, scale, distribute, and use; however, they are unable to provide quantitative information. To enable quantitative analysis of lateral flow immunoassay device results, microgating technology was used to develop an innovative spectrochip that can be integrated into a portable, palm-sized device that was capable of capturing high-resolution reflectance spectrum data for quantitative immunoassay diagnostics. Using predefined spiked concentrations of recombinant anti-SARS-CoV-2 immunoglobulin G (IgG), this spectrochip-coupled immunoassay provided extraordinary sensitivity, with a detection limit as low as 186 pg/mL. Furthermore, this platform enabled the detection of anti-SARS-CoV-2 IgG in all PCR-confirmed patients as early as day 3 after symptom onset, including two patients whose spectrochip tests would be regarded as negative for COVID-19 using a direct visual read-out without spectral analysis. Therefore, the quantitative lateral flow immunoassay with an exceptionally low detection limit for SARS-CoV-2 is of value. An increase in the number of patients tested with this novel device may reveal its true clinical potential.

## 1. Introduction

Since the first outbreak in China in 2019, coronavirus disease 2019 (COVID-19) has spread across continents. While its viral etiology and genome sequence were soon identified based on its genetic similarity to severe acute respiratory syndrome coronavirus (SARS-CoV) [1], the pandemic has resulted in over 104 million confirmed patients and 2,290,500 deaths as of 7 February 2021 [2]. To combat this pandemic, the development of COVID-19 vaccines has been making progress at remarkable speed. In December 2020, the U.S. Food and Drug Administration (FDA) and the European Medicines Agency (EMA) granted authorization for the emergency use of the messenger RNA (mRNA) vaccine developed by BioNTech and Pfizer. Soon afterward, a second mRNA vaccine developed by Moderna and the U.S. National Institute of Health (NIH) was also approved by FDA. Notably, although these vaccines have shown excellent efficacy at preventing symptomatic SARS-CoV-2 infection [3,4,5], global access to COVID-19 vaccination may not be achieved until 2022–2023. Moreover, it remains to be determined how effectively these vaccines can protect against new variants of SARS-CoV-2. Accordingly, rapid screening and strict quarantine measures remain a pivotal approach for limiting the pandemic.

To date, the two primary test types for diagnosing viral infections are reverse transcription polymerase chain reaction (RT-PCR) and IgM/IgG enzyme-linked immunosorbent assay (ELISA) [6,7,8,9]. Although RT-PCR is highly sensitive and remains the gold standard test for diagnosing SARS-CoV-2, it has several limitations. Specifically, the detection of viral nucleic acid using PCR requires sufficient quantities of high-quality viral RNA, which is sometimes difficult to obtain due to variances in the sampling technique, patient viral load, timing of infection, and onset of symptoms. In addition, RNA samples are vulnerable to degradation, and well-trained scientists must perform PCR using complex laboratory equipment [10,11]. Notably, these limitations are overcome by serology testing. Phlebotomy is less technique-sensitive and produces less sample variance as compared to nasopharyngeal or oropharyngeal swabbing for PCR testing. Serum antibodies are also more stable than viral RNA collected via swabbing. In addition, IgM is potentially useful for detecting recent infection for most cases because it usually becomes undetectable weeks following infection, and the presence of the IgG antibody often indicates a past infection because it generally does not appear until 7 to 10 days after the onset of infection and may last for months or years after infection [12,13,14]. Accordingly, serological tests offer some advantages to complement PCR-based diagnosis and screening for COVID-19. Notably, it has to be emphasized that not all COVID-19 patients will develop detectable IgM or IgG antibodies [15]; thus, PCR testing should always be performed for patients with negative serology test results that are suspected of COVID-19 infection.

Although serological and antibody-based assays, including ELISA and lateral flow immunoassays, are urgently needed, several challenges remain. An issue that limits the practicality of serological antibody testing as a primary tool for screening COVID-19 relates to seroconversion. Currently, most studies reported that, while the viral load peaked during the first week of infection, seroconversion started around days 3 to 10, and patients mostly tested positive for anti-viral IgG within 14 to 19 days after symptom onset [16,17,18]. In the early days of an infection, the immune response is evolving and virus-specific antibodies have not yet accumulated to levels that are detectable by most current serological test methods [19]. In addition, the individual immune responses may vary greatly across patients and most reports of antibody reaction are skewed toward severe and hospitalized cases [6]; therefore, it is not clear whether the immune reaction remains detectable in asymptomatic or presymptomatic patients. Moreover, it has been reported that certain patients with mild symptoms recovering from COVID-19 eventually did not develop SARS-CoV-2 antibodies, thus raising concern regarding the usefulness of serological antibody tests for COVID-19 screening [20]. Although it is possible that PCR gave false-positive results and the tested subjects were not actually infected with SARS-CoV-2, it is more likely that the magnitude of the antibody reaction was not detectable via currently available serological tests. Accordingly, an improved serological assay with an exceptionally low detection limit would certainly aid in the effort to control the current COVID-19 pandemic.

A lateral flow immunoassay (LFA) is a paper-based in situ detection platform that is characteristically inexpensive, easy to use, and can be operated by a non-health-care provider [21,22]. While LFA is an ideal point-of-care test, conventional colorimetric LFAs have unsatisfactory limits of detection (LODs) because the subtle color variations are difficult to differentiate visually. Nonetheless, the chromophores on the paper test strips emit complex spectra at various wavelengths that can be readily detected by a spectrometer to provide precise and quantifiable information. Thus, we proposed the coupling of an LFA with a portable spectrochip device to improve sensitivity and enable quantitative analysis. Additionally, we employed a gold colloid, which is a suspension of gold nanoparticles, to enhance the visibility and stability of the LFA. We believe that the extraordinary detection limit that is achievable with this new platform (i.e., the integration of the immunoassay and the optical reader) may make it an invaluable tool for accurate serological COVID-19 antibody testing.

## 2. Results

### 2.1. The Spectrum Analyzer System Provides High-Performance Spectral Analysis for the COVID-19 IgM/IgG Test Strip

**Figure 1:**
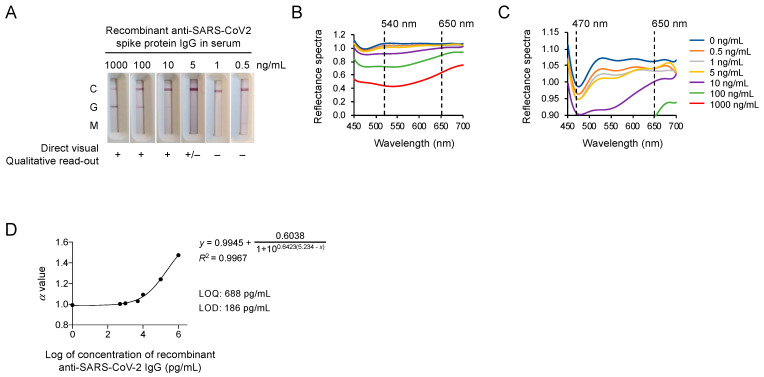
The spectrum analyzer provided an excellent detection limit. (**A**) The coronavirus disease 2019 (COVID-19) IgM/IgG test strips loaded with predetermined amounts of spiked IgG. (**B**) The reflectance spectra of the predetermined amounts of spiked IgG in a standard scale. (**C**) The reflectance spectra of the predetermined amounts of spiked IgG in an amplified scale. (**D**) Four-parameter logistic (4-PL) curve fit for the *α* value of the anti-SARS-CoV-2 IgG at the concentrations of 0.5, 1, 5, 10, 100, and 1000 ng/mL. The error bar that indicates the standard deviation was included but was too small to be visible.

In this quantitative platform system, the color intensity of an LFA test is analyzed using the spectrometer, where the presence of the COVID-19 IgG or IgM is based on a cut-off value for the spectral intensity. Thus, the LOD needs to be determined for evaluating the performance of this platform system. To this end, COVID-19 IgM/IgG test strips loaded with various predetermined, spiked amounts of IgG in serum (0, 0.5, 1, 5, 10, 100, 1000 ng/mL) were used to obtain the continuous reflectance spectra (from wavelengths 450 to 700 nm), where the data from three replicates were combined for the analysis. for analysis. As shown in Figure 1A, the presence of a band at the control (C) line on all test strips validated the tests of various IgG concentrations. Because we loaded IgG, and not IgM, there was no band at the IgM (M) line on each test strip. The band intensities at the IgG (G) line decreased as the concentration of spiked IgG decreased, and the bands became nearly invisible on the test strips that had been loaded with the IgG at concentrations lower than 5 ng/mL. The percentage of reflectance spectra reversely correlated with the amount of antibody complexes and, therefore, were decreased as the IgG concentration increased (Figure 1B). Notably, we found that the reflectance spectra of IgG at high concentrations (10, 100, 1000 ng/mL) were well separated at 540 nm and the reflectance spectra derived from low IgG concentrations (<5 ng/mL) were still clearly differentiated at 470 nm after amplifying the reflectance spectra scale (Figure 1C). The reflectance spectra were used to acquire an *α* value for constructing an IgG concentration standard curve of reflectance. As shown in Figure 1D, the four-parameter logistic regression fit the reflectance spectra data the best, providing an *R*^2^ value of 0.9967. Based on this regression model, the LOD and limit of quantification (LOQ), which were three and ten standard deviations from the average blank value, were determined as 186 pg/mL and 688 pg/mL, respectively. Therefore, this platform indeed provided excellent sensitivity for detecting a trace amount of SARS-CoV-2 antibodies.

### 2.2. The New Spectrum Analyzer System May Enable the Identification of Patients That Would Otherwise Produce False Negative Test Results

**Figure 2:**
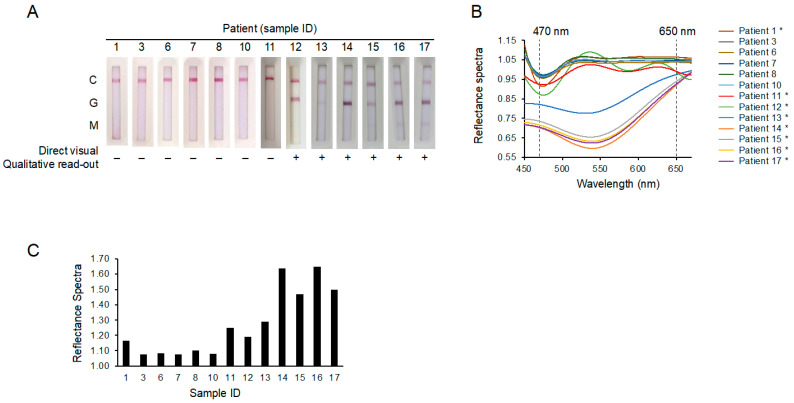
The excellent limit of detection prevented false-negative COVID-19 test results. (**A**) The results of the COVID-19 IgM/IgG test strips were used to test a panel of 13 suspected patients. Only patient ID#s 12, 13, 14, 15, 16, and 17 were clearly considered positive using a direct visual qualitative read-out. (**B**) The percentage of reflectance spectra derived from the patients’ test strips. Stars mark those patients who tested positive according to the spectrochip analysis. (**C**) The *α* value of patients’ reflectance spectra at 470 nm vs. 650 nm.

To determine whether this COVID-19 IgG/IgM test platform was of clinical value, blood samples from 13 suspected patients collected as early as day 3 after the onset of symptoms were blind tested. As shown in Figure 2A, the appearance of C lines validated the tests, but the lack of visible G lines on the first seven test strips (patient ID#s 1, 3, 6, 7, 8, 10, and 11) without spectral analysis implied that these patients tested negative for COVID-19. Positive G lines were clearly seen on test strips from patient ID#s 12, 13, 14, 15, 16, and 17. However, with the use of the new spectrum analyzer system for spectral analysis, we found that the *α* values of another two patients (ID#s 1 and 11) were also higher than those of the other patients (ID#s 3, 6, 7, 8, 9, and 10) (Figure 2B,C). Quantitative analysis revealed that the IgG levels in the sample from these eight patients (ID#s 1, 11, 12, 13, 14, 15, 16, and 17) were detectable (above the LOD of 186 pg/mL), whereas the IgG levels in the remaining patient samples were all below the detection limit (Table 1). Importantly, RT-PCR testing confirmed our findings regarding the patients who were tested SARS-CoV-2 IgG positive using this approach. This result suggests that the new spectrum analyzer system may be able to identify the potentially infected patients who would otherwise produce negative test results via a serological assay alone.

## 3. Discussion

More than one year after the start of the COVID-19 outbreak, countries around the globe still face many difficulties. In some areas, the number of patients to be tested overwhelms the testing capacity. Furthermore, while COVID-19 was initially diagnosed in the presence of pneumonia symptoms, the adequacy of symptom-based screening is questioned by an increasing number of asymptomatic or presymptomatic infections [23,24] and the actual prevalence of COVID-19 is likely underestimated. Accordingly, serological assays that can be widely distributed and detect present/past infections are very much in need. Among an array of potential serological test methods, LFAs are ideal for large-scale screening and point-of-care testing. However, the read-out is primarily qualitative (yes or no) and is generally not considered sensitive enough for diagnosing serious infections, such as COVID-19. In an effort to provide a sensitive quantitative capacity to the LFA, we integrated a newly designed spectrochip into our COVID-19 test strip procedures. This analytical platform may help to identify the infected patients who have not produced strong antibody responses.

A low detection limit for assays is critical for medical diagnostics. SARS-CoV-2 is highly infectious and potentially deadly for certain patients, and insufficient detection limits may lead to false-negative test results. For PCR-based diagnostic tests that have been approved for Emergency Use Authorization (EUA) by the U.S. FDA, the detection limits of the viral RNA range from 40 to 100,000 copies/mL [6,25]. Meanwhile, viral RNA often becomes detectable as early as day 1 after the onset of infection and peaks within the first week of symptom onset. However, the targets of serological assays, primarily IgM or IgG antibodies, often begin to increase 7 to 10 days after the onset of symptoms [6]. Notably, in this study, we showed that LFAs may possibly detect SARS-CoV-2 infection as early as 3 days after symptom onset. This implies that the antibody reaction to initial infection may be detectable at an earlier time point than previously assumed. As such, a lateral flow immunoassay system with an improved LOD may provide great clinical impact.

While the LFA with a low LOD may allow for the identification of a weak antibody response, it has to be emphasized that both the LOD and LOQ were obtained through the relevant numerical calculations (mean of blank plus 3- or 10-fold the standard deviation) and would be affected by the errors associated with the calibration curve fitting. Therefore, the assessment of test results based on the provided LOD and LOQ should be done carefully. Nonetheless, as a false negative result is more consequential than a false-positive result, a low detection limit is still of great value to alert healthcare professionals of a potential infection. 

To date, there are several chemiluminescent immunoassay analyzers that are available for COVID-19 molecular diagnostics, among which, Abbott, Bio-Rad, and Roche are the top providers to receive EUA for commercial tests to diagnose SARS-CoV-2 infection. While the Abbott chemiluminescent microparticle immunoassay (CMIA), Bio-Rad ELISA, and Roche electrochemiluminescence immunoassays (ECLIA) kits use laboratory-based processes and materials, our platform uses LFA and does not require extra chemical reagents for sample preparation by medical professionals. In addition, these systems use the spectrum between 300 and 800 nm and a 15 nm sampling interval, whereas our spectrochip platform uses the entire (300–1000 nm) spectrum and a sampling interval of 3–5 nm to scan the test strips, thus providing a higher spectral analysis resolution (Table 2) [26,27,28,29]. Furthermore, the gold colloid incorporated into this LFA appeared in red (for spherical particles smaller than 100 nm) or blue/purple (for larger spherical particles), thus further augmenting the diagnostic value of this colorimetric approach. Moreover, gold nanoparticles have a high affinity to sulfhydryl (−SH) groups and are thus readily conjugated to various proteins, including antibodies. The use of gold nanoparticles can also improve the stability of antigens and prolong shelf life. Hence, our platform is not only easy to use but also highly sensitive for detecting an antibody response to COVID-19. Notably, the gold colloid can be substituted with fluorescent cadmium telluride quantum dots, which has been found to provide a LOD at the pg/mL level. However, the detection of a fluorescent LFA requires a specialized fluorescence reader and storage requirements to strictly avoid light. Because of these limitations, fluorescent LFA is less well suited for use in home-based or point-of-care tests.

While the presented data are promising, this study was nonetheless limited to a small sample size (13 patients in this study). It is not uncommon to have some patients report positive after multiple consecutive negative PCR testing. Although this did not occur in our study, an implication of this finding is that a definitive diagnosis of SARS-CoV-2 infection will be uncertain for some cases, and therefore, a larger test sample size is critical to evaluate the efficacy of this new platform.

**Figure 3:**
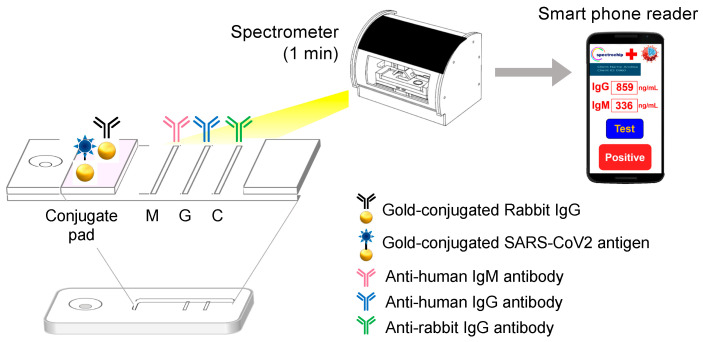
The workflow of this new spectrum analyzer platform system involves applying 1 mL of whole blood, blood serum, or plasma from a fingertip or from a vein to the test strip and provides results in 10–15 min depending on the reagent used. The test strip is placed in a spectrometer for quantitative spectral analysis. This scan takes approximately one minute to complete. Automatic scanning of the rapid test strip is activated with an app. Full-spectrum antibody reflex optical signals are acquired from the spectral optical module to analyze the COVID-19 IgG/IgM full-spectrum antibody distribution and concentration with standard quantification. The results can be used in conjunction with clinical timetables to analyze and track the spread of COVID-19.

In conclusion, this study showed that, with the aid of a newly designed spectrochip, a lateral flow immunoassay could be as sensitive as the majority of COVID-19 diagnostics on the market. A schematic of the workflow for this spectrum analyzer platform is provided in Figure 3. Hopefully, this new platform may fill the gap in current diagnostic capacity and contribute to preparations for a gradual return to pre-pandemic conditions in a post-pandemic world.

## 4. Methods

### 4.1. Patients and Samples

A total of 13 patients admitted to Kaohsiung Municipal Siaogang Hospital, Taiwan, based on symptoms of acute respiratory infection syndrome were enrolled in this study after providing informed consent (Table 3). These patients were tested for SARS-CoV-2 infection using real-time PCR on samples from nasopharyngeal or oropharyngeal swabs. Serum samples were also collected from each patient on day 3 after symptom onset.

### 4.2. Statement

All experiments and methods were performed in accordance with relevant guidelines and regulations. Informed consent for the publication of identifying information in an online open-access publication in the methods section was also obtained from all subjects. All experimental protocols, including blood collection, the spectrochip test, and the PCR assay, were approved by the Municipal Siaogang Hospital and Kaohsiung Medical University Hospital Committee (Institutional Review Board (IRB) No.: KMUHIRB-F(I)-20200044).

### 4.3. Lateral Flow Immunoassays

The Biomedomics SARS-CoV-2 rapid test strip (BioMedomics, Morrisville, NC 27560, USA) was used in this study because it was one of a few LFAs for COVID-19 that were commercially available with U.S. FDA EUA approval during April 2020. This nitrocellulose test strip used colloidal gold-conjugated antibodies to detect human IgG and IgM antibodies. The optimization test was performed to determine the most appropriate combination of the quantity of SARS-CoV-2 nuclear protein and gold nanoparticle size for conjugation, which was based on the colorimetric analysis of each combination. The test strip is pre-coated with capture reagents by spraying it to create perpendicular lines of recombinant viral SARS-CoV-2 protein conjugated with 15 nm diameter gold colloids. This nitrocellulose strip is also coated with mouse anti-human IgG monoclonal antibody at the G line, as well as the same amount of the quality control antibody at the C line. As the applied sample flows down the strip, anti-SARS-CoV-2 IgG and/or IgM antibodies present in the sample form antibody complexes with the colloidal gold-labeled recombinant SARS-CoV-2 antigen. These complexes are then captured in the perpendicular G and/or M lines of the test strip, where they are presented as colored bands.

### 4.4. Reflectance Spectral Analysis

The LFA spectrum analyzer (ONE InstantCare chromogenic rapid screening analyzer, Spectrochip Inc., Hsinchu 300, Taiwan; Taiwan FDA: MD (I)-008090 and U.S. FDA: 3017810861) is equipped with the cassette that is designed for the Biomedomics COVID-19 rapid test to detect the reflectance spectrum from IgM/IgG antibodies of the test strips. The spectrum analyzer provides a continuous spectrum and captures a high-resolution reflectance spectrum of the test lines (M or G lines) on a test strip via an optical module, where the optical signal of reflectance spectra is analyzed by the spectrum reader. This spectrum reader provides high-resolution (3–5 nm) results across a vast spectral range (300 to 1100 nm). The primary reflectance wavelengths detected by using this spectrum reader are 540 nm and 470 nm (for samples with a low IgG concentration), with the main reference wavelength being 650 nm. The *α* value was calculated using the ratio of the spectra at the minimum reflectance and reference wavelengths:

$$\alpha =\frac{\mathrm{Reflectance}\;\mathrm{at}\;650\;\mathrm{nm}}{\mathrm{Reflectance}\;\mathrm{at}\;540\;\mathrm{nm}\;\mathrm{or}\;470\;\mathrm{nm}}.$$

The *α* value refers to the color reflection value of the optical scanning antibody rapid screening test piece. A higher *α* value indicates a stronger reflection color intensity of the colloidal gold antibody-conjugated IgG and IgM complexes, which indicates a higher antibody concentration. 

### 4.5. Limit of Detection and Limit of Quantification

LOD = Blank (mean) + 3 × Blank (standard deviation) → Fit to the Figure D equation
LOQ = Blank (mean) + 10 × Blank (standard deviation) → Fit to the Figure D equation

The LOD and LOQ were estimated from the mean of the blank *α* value, the standard deviation of the blank *α* value, the slope (analytical sensitivity) of the calibration plot, and a defined confidence factor, using the following values: the mean of the blank *α* value was 0.99787, the standard deviation of the blank *α* value was 0.00136, the CV of the blank (%) was 0.14%, and the 95% confidence interval was 98.5–100%. 

The LOD was estimated using the mean of the blank *α* value plus 3 times the standard deviation of the mean of the blank *α* value for the calibration plot using the following equation:

$$y=0.9945+\frac{0.6038}{1+10^{0.6423\left(5.234\;-\;x\right)}}$$

where *y* is the *α* value and *x* is the log of the IgG concentration (pg/mL). Accordingly, the *α* value of the LOD was equal to 1.00195, the log of the LOD concentration (pg/mL) was equal to 2.27039, and the LOD concentration was 186 pg/mL. The same calculation was applied to find the LOQ.

## Figures and Tables

**Table 1:** Summary of results of a spectrochip and PCR test of enrolled patients.

Sample ID	Days from Onset to PCR	Spectrochip Direct Visual Read-Out	Spectrochip Quantitative Estimate (pg/mL)	Spectrochip Results	PCR Results
1	3	Negative	191	Weakly positive	Positive
3	0	Negative	162	Negative	Negative
6	1	Negative	165	Negative	Negative
7	12	Negative	162	Negative	Negative
8	5	Negative	170	Negative	Negative
10	15	Negative	164	Negative	Negative
11	18	Negative	379	Positive	Positive
12	10	Positive	499	Positive	Positive
13	24	Positive	578	Positive	Positive
14	13	Positive	1263	Positive	Positive
15	10	Positive	952	Positive	Positive
16	33	Positive	1282	Positive	Positive
17	43	Positive	2481	Positive	Positive

PCR: polymerase chain reaction.

**Table 2:** Summary of the age, gender, symptoms, and the date of the PCR and spectrochip testing for the 13 enrolled patients.

Sample ID	Age	Gender	Date of Symptom Onset	Symptoms	Date of PCR Test	PCR Result	Date of Blood Draw	Date of Spectrochip Test
1	31	M	14/3/2020	Stuffy running nose, cough, sore throat	17/3/2020	Confirmed	17/3/2020	8/4/2020
3	35	M	10/3/2020	Sore throat	10/3/2020	Negative	10/3/2020	8/4/2020
6	23	F	11/3/2020	Fever, headache, sore throat	12/3/2020	Negative	12/3/2020	8/4/2020
7	55	F	29/2/2020	Cough, sore throat	12/3/2020	Negative	12/3/2020	8/4/2020
8	41	F	9/3/2020	Headache, fever, body aches and rash	14/3/2020	Negative	16/3/2020	8/4/2020
10	48	M	12/3/2020	Chest pain, chest tightness, short of breath	27/3/2020	Negative	30/3/2020	8/4/2020
11	31	M	14/3/2020	Stuffy running nose, cough, sore throat	24/3/2020	Confirmed	1/4/2020	8/4/2020
12	31	M	14/3/2020	Stuffy running nose, cough, sore throat	1/4/2020	Confirmed	24/3/2020	8/4/2020
13	27	F	6/3/2020	Fever, cough, abnormal taste/smell, rhinorrhea	18/3/2020	Confirmed	30/3/2020	1/9/2020
14	50	F	24/3/2020	Fever, abnormal taste, chills	31/3/2020	Confirmed	6/4/2020	1/9/2020
15	23	F	27/3/2020	Abnormal smell, rhinorrhea	27/3/2020	Confirmed	6/4/2020	1/9/2020
16	21	F	19/3/2020	Fever, cough, abnormal taste/smell, diarrhea, chest pain	24/3/2020	Confirmed	21/4/2020	1/9/2020
17	34	M	10/3/2020	Fever, cough, abnormal taste/smell	18/3/2020	Confirmed	21/4/2020	1/9/2020

**Table 3:** The comparison of commercially available chemiluminescent immunoassay kits and analyzers for COVID-19 diagnostics.

COVID-19 Molecular Diagnostics Provider	New Spectrum Analyzer Platform	Abbott CMIA	Bio-Rad ELISA	Roche ECLIA
Platform	LFA	Laboratory-based inventories	Laboratory-based inventories	Laboratory-based inventories
Spectral analysis	Reflection spectra (*α* light intensity) (300–1000 nm) Resolution = 5 nm	Luminescence COI (300–800 nm)	Filter (titer) (OD = 450 nm, ref = 650 nm)	Relative light unit (specific 300–650 nm) Resolution = 15 nm
Sensitivity (confirmed cases/test positive cases)	100% (8/8)	89% (109/122) [30]	98% (49/50) [31]	82% (409/496) [32]
Sample preparation	No	Yes	Yes	Yes
Specimen	Whole blood, Serum, plasma	Serum, plasma	Serum, plasma	Serum, plasma
Calculation	Index (*α*)	Index (S/C)	Information not available	Information not available
Turnaround time	5–10 min	10–15 min	1 h	1 h

CMIA: chemiluminescent microparticle immunoassay, COI: cut-off index, ECLIA: electrochemiluminescence immunoassays, ELISA: enzyme-linked immunosorbent assay, LFA: lateral flow immunoassay, OD: optical density.
